# Supplementary material for: Direct aortic route versus transaxillary route for transcatheter aortic valve replacement: a systematic review and meta-analysis
Source: PeerJ. 2020 May 12;8:e9102. doi: 10.7717/peerj.9102 (PMC7227658; doi:10.7717/peerj.9102)
Supplement: Supplemental Information 1 [file peerj-08-9102-s001.docx]

**Supplementary Material 1**. The exact string of keywords used to do the search in each database.

**Cochrane:**

((Transcatheter AND "aortic valve") OR TAVR OR TAVI) AND (Transaortic OR "direct aortic" OR transaxillary OR axillary OR trans-subclavian OR subclavian)

**Embase:**

#1 (transcatheter AND 'aortic valve') OR TAVI OR TAVR

#2 'direct aortic' OR transaortic OR subclavian OR 'trans subclavian' OR axillary OR transaxillary

#3 #1 AND #2

**Ovid Medline:**

表單的頂端

| 1 | ((transcather and "aortic valve") or TAVR or TAVI).mp. [mp=title, abstract, original title, name of substance word, subject heading word, floating sub-heading word, keyword heading word, organism supplementary concept word, protocol supplementary concept word, rare disease supplementary concept word, unique identifier, synonyms] |
| --- | --- |
| 2 | ("direct aortic" or transaortic or trans-subclavian or subclavian or transaxillary or axillary).mp. [mp=title, abstract, original title, name of substance word, subject heading word, floating sub-heading word, keyword heading word, organism supplementary concept word, protocol supplementary concept word, rare disease supplementary concept word, unique identifier, synonyms] |
| 3 | 1 and 2 |

表單的底部

表單的頂端

表單的底部

**Pubmed:**((((((“direct aortic”) OR (transaortic)) OR (transaxillary)) OR (axillary)) OR (subclavian)) OR (trans-subclavian)) AND ((((Transcatheter) AND (“aortic valve”)) OR (TAVR)) OR (TAVI))
Filters: **Full text, Journal Article, English**
