# Supplementary material for: Direct aortic route versus transaxillary route for transcatheter aortic valve replacement: a systematic review and meta-analysis
Source: PeerJ. 2020 May 12;8:e9102. doi: 10.7717/peerj.9102 (PMC7227658; doi:10.7717/peerj.9102)
Supplement: Supplemental Information 2 [file peerj-08-9102-s002.docx]

**Supplementary Material 2**. List of studies excluded because of using devices other than Edward or Medtronic after abstract screened (Baştuğ et al., 2016; Bushnaq et al., 2016; Deuschl et al., 2017; Kasapkara et al., 2016).

Baştuğ S, Aslan AN, Sarı C, Süygün H, and Bozkurt E. 2016. First trans-subclavian transcatheter aortic valve replacement using Lotus valve system. *Turk Kardiyoloji Dernegi arsivi : Turk Kardiyoloji Derneginin yayin organidir* 44:507-510. 10.5543/tkda.2015.81242

Bushnaq H, Metz D, Petrov A, Frantz S, Hofmann U, Raspé C, and Treede H. 2016. Direct aortic access for transcatheter aortic valve replacement with a fully repositionable and retrievable nonmetallic valve system. *The Journal of thoracic and cardiovascular surgery* 152:1611-1615. 10.1016/j.jtcvs.2016.06.063

Deuschl F, Schofer N, Seiffert M, Hakmi S, Mizote I, Schaefer A, Schirmer J, Reichenspurner H, Blankenberg S, Conradi L, and Schäfer U. 2017. Direct percutaneous transaxillary implantation of a novel self-expandable transcatheter heart valve for aortic stenosis. *Catheterization and Cardiovascular Interventions* 90:1167-1174. 10.1002/ccd.26986

Kasapkara HA, Aslan AN, Ayhan H, Baştuğ S, Süygün H, Keleş T, Durmaz T, and Bozkurt E. 2016. Trans-subclavian aortic valve replacement with various bioprosthetic valves: Single-center experience. *Turk Kardiyoloji Dernegi arsivi : Turk Kardiyoloji Derneginin yayin organidir* 44:582-589. 10.5543/tkda.2016.45774
