# Supplementary material for: Direct aortic route versus transaxillary route for transcatheter aortic valve replacement: a systematic review and meta-analysis
Source: PeerJ. 2020 May 12;8:e9102. doi: 10.7717/peerj.9102 (PMC7227658; doi:10.7717/peerj.9102)
Supplement: Supplemental Information 3 [file peerj-08-9102-s003.docx]

1. Rationale for conducting the meta-analysis:

The transfemoral (TF) route is used as a default for performing transcatheter aortic valve replacement (TAVR) in many institutions. However, peripheral vascular occlusion, stenosis, calcification, or tortuosity precludes TF access in approximately 5%–10% of patients, necessitating the use of an alternative route. Transaxillary (TAx) and direct aortic (DAo) routes are the principal nonfemoral TAVR routes, but few studies have compared their outcomes. Therefore, we conducted this systematic review and meta-analysis to compare the morbidity and mortality associated with TAx and DAo TAVR.

1. The contribution that the meta-analysis makes to knowledge in light of previously published related reports, including other meta-analyses and systematic reviews.

With nearly 3,000 patients included, our study is the largest sample used thus far for comparing TAx and DAo TAVR outcomes. We found that 30-day and 1-year mortality rates were similar between the two groups, but postoperative stroke, permanent pacemaker implantation and valve malposition rates were significantly higher in patients who underwent TAx TAVR. The present meta-analysis provides valuable information for clinicians when choosing alternative routes for TAVR candidates who are not suitable for TF route.
