# Supplementary material for: Direct aortic route versus transaxillary route for transcatheter aortic valve replacement: a systematic review and meta-analysis
Source: PeerJ. 2020 May 12;8:e9102. doi: 10.7717/peerj.9102 (PMC7227658; doi:10.7717/peerj.9102)
Supplement: Supplemental Information 5 [file peerj-08-9102-s005.docx]

**Supplementary Table 2**. Studies excluded after full-text reviewed, with the reasons and the reference.

| Reasons for exclusion | Articles (author, year) | n |
| --- | --- | --- |
| Case number < 10 | (Tayal et al., 2017) | 1 |
| Combined CABG | (Kobayashi et al., 2016) | 1 |
| Devices other than Medtronic or Edward were used | (Amrane et al., 2014) (Ciuca et al., 2016) (McCarthy et al., 2017) (Anselmi et al., 2018a) | 4 |
| Duplicated cohort | (Abu Saleh et al., 2015; Arai et al., 2016; Bapat et al., 2016; Bapat et al., 2012; Bonaros et al., 2017; Bruschi et al., 2012; Hayashida et al., 2013; Lardizabal et al., 2013) (Blackman et al., 2014; Cioni et al., 2011; Petronio et al., 2012; Petronio et al., 2010; Saia et al., 2013; Schäfer et al., 2012; Taramasso et al., 2011) (Godino et al., 2010; Suri et al., 2015) (Romano et al., 2017) (Bonaros et al., 2018) | 19 |
| No outcome data of TAx or DAo TAVR | (Ahad et al., 2017; Basir et al., 2016; Blackman et al., 2014; Dusse et al., 2016; Herrmann et al., 2016; Kiefer et al., 2015; Kodali et al., 2016; Koh et al., 2015; Patel et al., 2016; Salizzoni et al., 2016; Sannino et al., 2016; Scherner et al., 2015; Thourani et al., 2013; Watanabe et al., 2013; Wenaweser et al., 2014) (Kalra et al., 2016; Kochman et al., 2016; Lange et al., 2011; Pascual et al., 2013) (Gilard et al., 2016; Gotzmann et al., 2013; Iung et al., 2014; Parma et al., 2017; Sherif et al., 2014; Sinning et al., 2012) (Bittar & Castilho, 2017; Dodson et al., 2017; Jarrett et al., 2017; Kawaguchi et al., 2017; Lucchese, Montarello & Bapat, 2017; Mathur et al., 2017) (Alkhalil et al., 2018; Armoiry et al., 2018; Azevedo et al., 2018; Barbanti et al., 2018; Bavishi et al., 2018; Beurtheret et al., 2019; Beve et al., 2019; Campelo-Parada et al., 2018; Chamandi et al., 2018; Chopra, Tong & Yakubov, 2018; Deharo et al., 2018; Elbaz-Greener et al., 2018; Hall et al., 2019; Hellhammer et al., 2018; Porterie et al., 2019; Rogers et al., 2018) | 47 |
| Not using VARC definition | (Bapat, Attia & Thomas, 2012; Clarke et al., 2013; Cocchieri et al., 2016; Henn et al., 2017; Thourani et al., 2015) (Kasapkara et al., 2016; López-Otero et al., 2011; Modine et al., 2011; Modine et al., 2012) (Fröhlich et al., 2015; Reardon et al., 2014) (O'Hair et al., 2018; Stańska et al., 2018) | 13 |
| Includes the valve-in-valve procedure | (Anselmi et al., 2018b) | 1 |

References:

Abu Saleh WK, Goswami R, Chinnadurai P, Al Jabbari O, Barker CM, Lin CH, Kleiman N, Reardon MJ, and Ramlawi B. 2015. Direct Aortic Access Transcatheter Aortic Valve Replacement: Three-Dimensional Computed Tomography Planning and Real-Time Fluoroscopic Image Guidance. *Journal of Heart Valve Disease* 24:420-425.

Ahad S, Wachter K, Rustenbach C, Stan A, Hill S, Schaufele T, Ursulescu A, Franke UF, and Baumbach H. 2017. Concomitant therapy: off-pump coronary revascularization and transcatheter aortic valve implantation. *Interact Cardiovasc Thorac Surg*. 10.1093/icvts/ivx029

Alkhalil A, Lamba H, Deo S, Bezerra HG, Patel SM, Markowitz A, Simon DI, Costa MA, Davis AC, and Attizzani GF. 2018. Safety of shorter length of hospital stay for patients undergoing minimalist transcatheter aortic valve replacement. *Catheterization and cardiovascular interventions : official journal of the Society for Cardiac Angiography & Interventions* 91:345-353. 10.1002/ccd.27230

Amrane H, Porta F, Van Boven AJ, Boonstra PW, Hofma SH, Head SJ, and Kappetein AP. 2014. Transcatheter aortic valve implantation using a direct aortic approach: A single-centre Heart Team experience. *Interact Cardiovasc Thorac Surg* 19:777-781. 10.1093/icvts/ivu247

Anselmi A, Dachille A, Auffret V, Harmouche M, Roisne A, Bedossa M, Le Breton H, and Verhoye J-P. 2018a. Evolution of Length of Stay After Surgical and Transcatheter Aortic Valve Implantation Over 8 Years in 1,849 Patients >75 Years of Age and Comparison Between Transfemoral and Transsubclavian Transcatheter Aortic Valve Implantation. *The American journal of cardiology* 122:1387-1393. 10.1016/j.amjcard.2018.06.051

Anselmi A, Tomasi J, Giardinelli F, Bedossa M, Rosier S, and Verhoye JP. 2018b. Safety and effectiveness of the transsubclavian approach for transcatheter aortic valve implantation with the 14-F CoreValve Evolut R device. *Journal of Cardiovascular Medicine* 19:664-668. 10.2459/JCM.0000000000000706

Arai T, Romano M, Lefevre T, Hovasse T, Bouvier E, Morice MC, Farge A, Garot P, and Chevalier B. 2016. Impact of procedural volume on outcome optimization in transaortic transcatheter aortic valve implantation. *Int J Cardiol* 223:292-296. 10.1016/j.ijcard.2016.07.184

Armoiry X, Obadia J-F, Pascal L, Polazzi S, and Duclos A. 2018. Comparison of transcatheter versus surgical aortic valve implantation in high-risk patients: A nationwide study in France. *The Journal of thoracic and cardiovascular surgery* 156:1017-1025.e1014. 10.1016/j.jtcvs.2018.02.092

Azevedo FS, Correa MG, Paula DHG, Felix ADS, Belem LHJ, Mendes APC, Silva VG, Marques BM, Monteiro AJdO, Weksler C, Colafranceschi AS, and Kasal DAB. 2018. Transcatheter Aortic Valve Replacement: The Experience of One Brazilian Health Care Center. *Brazilian journal of cardiovascular surgery* 33:1-7. <https://dx.doi.org/10.21470/1678-9741-2017-0117>

Bapat V, Frank D, Cocchieri R, Jagielak D, Bonaros N, Aiello M, Lapeze J, Laine M, Chocron S, Muir D, Eichinger W, Thielmann M, Labrousse L, Rein KA, Verhoye JP, Gerosa G, Baumbach H, Bramlage P, Deutsch C, Thoenes M, and Romano M. 2016. Transcatheter Aortic Valve Replacement Using Transaortic Access: Experience From the Multicenter, Multinational, Prospective ROUTE Registry. *JACC Cardiovasc Interv* 9:1815-1822. 10.1016/j.jcin.2016.06.031

Bapat V, Khawaja MZ, Attia R, Narayana A, Wilson K, Macgillivray K, Young C, Hancock J, Redwood S, and Thomas M. 2012. Transaortic Transcatheter Aortic valve implantation using Edwards Sapien valve: a novel approach. *Catheterization & Cardiovascular Interventions* 79:733-740. <https://dx.doi.org/10.1002/ccd.23276>

Bapat VN, Attia RQ, and Thomas M. 2012. Distribution of calcium in the ascending aorta in patients undergoing transcatheter aortic valve implantation and its relevance to the transaortic approach. *JACC: Cardiovascular Interventions* 5:470-476. <https://dx.doi.org/10.1016/j.jcin.2012.03.006>

Barbanti M, Costa G, Zappulla P, Todaro D, Picci A, Rapisarda G, Di Simone E, Sicuso R, Buccheri S, Gulino S, Pilato G, La Spina K, D'Arrigo P, Valvo R, Indelicato A, Giannazzo D, Imme S, Tamburino C, Patane M, Sgroi C, Giuffrida A, Trovato D, Monte IP, Deste W, Capranzano P, Capodanno D, and Tamburino C. 2018. Incidence of Long-Term Structural Valve Dysfunction and Bioprosthetic Valve Failure After Transcatheter Aortic Valve Replacement. *Journal of the American Heart Association* 7:e008440. <https://dx.doi.org/10.1161/JAHA.117.008440>

Basir MB, Velez C, Fuller B, Wyman J, Paone G, Wang DD, Guerrero M, Greenbaum A, and O'Neill W. 2016. Rates of vascular access use in transcatheter aortic valve replacement: A look into the next generation. *Catheterization and Cardiovascular Interventions* 87:E166-E171. 10.1002/ccd.26116

Bavishi C, Kolte D, Gordon PC, and Abbott JD. 2018. Transcatheter aortic valve replacement in patients with severe aortic stenosis and heart failure. *Heart failure reviews* 23:821-829. 10.1007/s10741-018-9726-8

Beurtheret S, Karam N, Resseguier N, Houel R, Modine T, Folliguet T, Chamandi C, Com O, Gelisse R, Bille J, Joly P, Barra N, Tavildari A, Commeau P, Armero S, Pankert M, Pansieri M, Siame S, Koning R, Laskar M, Le Dolley Y, Maudiere A, Villette B, Khanoyan P, Seitz J, Blanchard D, Spaulding C, Lefevre T, Van Belle E, Gilard M, Eltchaninoff H, Iung B, Verhoye JP, Abi-Akar R, Achouh P, Cuisset T, Leprince P, Marijon E, Le Breton H, and Lafont A. 2019. Femoral Versus Nonfemoral Peripheral Access for Transcatheter Aortic Valve Replacement. *Journal of the American College of Cardiology* 74:2728-2739. 10.1016/j.jacc.2019.09.054

Beve M, Auffret V, Belhaj Soulami R, Tomasi J, Anselmi A, Roisne A, Boulmier D, Bedossa M, Leurent G, Donal E, Le Breton H, and Verhoye JP. 2019. Comparison of the Transarterial and Transthoracic Approaches in Nontransfemoral Transcatheter Aortic Valve Implantation. *American Journal of Cardiology* 123:1501-1509. 10.1016/j.amjcard.2019.01.040

Bittar E, and Castilho V. 2017. The cost of transcatheter aortic valve implantation according to different access routes. *Revista da Escola de Enfermagem da U S P* 51:e03246. <https://dx.doi.org/10.1590/S1980-220X2016050503246>

Blackman DJ, Baxter PD, Gale CP, Moat NE, Maccarthy PA, Hildick-Smith D, Trivedi U, Cunningham D, De Belder MA, and Ludman PF. 2014. Do Outcomes from transcatheter aortic valve implantation vary according to access route and valve type? the UK TAVI registry. *Journal of Interventional Cardiology* 27:86-95. 10.1111/joic.12084

Bonaros N, Kofler M, Frank D, Cocchieri R, Jagielak D, Aiello M, Lapeze J, Laine M, Chocron S, Muir D, Eichinger W, Thielmann M, Labrousse L, Bapat V, Rein KA, Verhoye J-P, Gerosa G, Baumbach H, Deutsch C, Bramlage P, Thoenes M, and Romano M. 2018. Balloon-expandable transaortic transcatheter aortic valve implantation with or without predilation. *The Journal of thoracic and cardiovascular surgery* 155:915-923. 10.1016/j.jtcvs.2017.10.071

Bonaros N, Petzina R, Cocchieri R, Jagielak D, Aiello M, Lapeze J, Laine M, Chocron S, Muir D, Eichinger W, Thielmann M, Labrousse L, Bapat V, Arne Rein K, Verhoye JP, Gerosa G, Baumbach H, Kofler M, Bramlage P, Deutsch C, Thoenes M, Frank D, and Romano M. 2017. Transaortic transcatheter aortic valve implantation as a first-line choice or as a last resort? An analysis based on the ROUTE registry. *Eur J Cardio-Thorac Surg* 51:919-926.

Bruschi G, de Marco F, Botta L, Cannata A, Oreglia J, Colombo P, Barosi A, Colombo T, Nonini S, Paino R, Klugmann S, and Martinelli L. 2012. Direct aortic access for transcatheter self-expanding aortic bioprosthetic valves implantation. *Annals of Thoracic Surgery* 94:497-503. <https://dx.doi.org/10.1016/j.athoracsur.2012.04.021>

Campelo-Parada F, Nombela-Franco L, Urena M, Regueiro A, Jiménez-Quevedo P, Del Trigo M, Chamandi C, Rodríguez-Gabella T, Auffret V, Abdul-Jawad Altisent O, DeLarochellière R, Paradis J-M, Dumont E, Philippon F, Pérez-Castellano N, Puri R, Macaya C, and Rodés-Cabau J. 2018. Timing of Onset and Outcome of New Conduction Abnormalities Following Transcatheter Aortic Valve Implantation: Role of Balloon Aortic Valvuloplasty. *Revista espanola de cardiologia (English ed)* 71:162-169. 10.1016/j.rec.2017.04.010

Chamandi C, Abi-Akar R, Rodés-Cabau J, Blanchard D, Dumont E, Spaulding C, Doyle D, Pagny JY, DeLarochellière R, Lafont A, Paradis JM, Puri R, Karam N, Maes F, Rodriguez-Gabella T, Chassaing S, Le Page O, Kalavrouziotis D, and Mohammadi S. 2018. Transcarotid compared with other alternative access routes for transcatheter aortic valve replacement. *Circulation: Cardiovascular Interventions* 11. 10.1161/CIRCINTERVENTIONS.118.006388

Chopra N, Tong MS, and Yakubov SJ. 2018. Very late occurrence of complete heart block without preexisting atrioventricular conduction abnormalities: A rare complication after transaortic valvular replacement. *HeartRhythm Case Reports* 4:77-81. 10.1016/j.hrcr.2017.11.010

Cioni M, Taramasso M, Giacomini A, Montorfano M, Latib A, Colombo A, Alfieri O, and Maisano F. 2011. Transaxillary approach: Short- and mid-term results in a single-center experience. *Innovations: Technology and Techniques in Cardiothoracic and Vascular Surgery* 6:361-365. 10.1097/IMI.0b013e318248e9ed

Ciuca C, Tarantini G, Latib A, Gasparetto V, Savini C, Di Eusanio M, Napodano M, Maisano F, Gerosa G, Sticchi A, Marzocchi A, Alfieri O, Colombo A, and Saia F. 2016. Trans-subclavian versus transapical access for transcatheter aortic valve implantation: A multicenter study. *Catheter Cardiovasc Interv* 87:332-338. 10.1002/ccd.26012

Clarke A, Wiemers P, Poon KK, Aroney CN, Scalia G, Burstow D, Walters DL, and Tesar P. 2013. Early experience of transaortic TAVI--the future of surgical TAVI? *Heart Lung Circ* 22:265-269. <https://dx.doi.org/10.1016/j.hlc.2012.11.002>

Cocchieri R, Koh EY, Wollersheim LW, Meregalli PG, Bardai A, Bouma BJ, and De Mol BA. 2016. Transaortic aortic valve implantation in 100 patients: Follow-up to 3 years. *Innovations: Technology and Techniques in Cardiothoracic and Vascular Surgery* 11:106-111. 10.1097/IMI.0000000000000254

Deharo P, Jaussaud N, Grisoli D, Camus O, Resseguier N, Le Breton H, Auffret V, Verhoye JP, Koning R, Lefevre T, Van Belle E, Eltchaninoff H, Gilard M, Leprince P, Iung B, Lambert M, Collart F, and Cuisset T. 2018. Impact of Direct Transcatheter Aortic Valve Replacement Without Balloon Aortic Valvuloplasty on Procedural and Clinical Outcomes: Insights From the FRANCE TAVI Registry. *JACC Cardiovascular interventions* 11:1956-1965. 10.1016/j.jcin.2018.06.023

Dodson JA, Williams MR, Cohen DJ, Manandhar P, Vemulapalli S, Blaum C, Zhong H, Rumsfeld JS, and Hochman JS. 2017. Hospital Practice of Direct-Home Discharge and 30-Day Readmission After Transcatheter Aortic Valve Replacement in the Society of Thoracic Surgeons/American College of Cardiology Transcatheter Valve Therapy (STS/ACC TVT) Registry. *Journal of the American Heart Association* 6:e006127. 10.1161/JAHA.117.006127

Dusse F, Edayadiyil-Dudásova M, Thielmann M, Wendt D, Kahlert P, Demircioglu E, Jakob H, Schaefer ST, and Pilarczyk K. 2016. Early prediction of acute kidney injury after transapical and transaortic aortic valve implantation with urinary G1 cell cycle arrest biomarkers. *BMC Anesthesiology* 16. 10.1186/s12871-016-0244-8

Elbaz-Greener G, Qiu F, Masih S, Fang J, Austin PC, Cantor WJ, Dvir D, Asgar AW, Webb JG, Ko DT, and Wijeysundera HC. 2018. Profiling Hospital Performance Based on Mortality After Transcatheter Aortic Valve Replacement in Ontario, Canada. *Circulation Cardiovascular quality and outcomes* 11:e004947-e004947. 10.1161/CIRCOUTCOMES.118.004947

Fröhlich GM, Baxter PD, Malkin CJ, Scott DJA, Moat NE, Hildick-Smith D, Cunningham D, Maccarthy PA, Trivedi U, De Belder MA, Ludman PF, and Blackman DJ. 2015. Comparative Survival after Transapical, Direct Aortic, and Subclavian Transcatheter Aortic Valve Implantation (Data from the UK TAVI Registry). *American Journal of Cardiology* 116:1555-1559. 10.1016/j.amjcard.2015.08.035

Gilard M, Eltchaninoff H, Donzeau-Gouge P, Chevreul K, Fajadet J, Leprince P, Leguerrier A, Lievre M, Prat A, Teiger E, Lefevre T, Tchetche D, Carrié D, Himbert D, Albat B, Cribier A, Sudre A, Blanchard D, Rioufol G, Collet F, Houel R, Dos Santos P, Meneveau N, Ghostine S, Manigold T, Guyon P, Grisoli D, Le Breton H, Delpine S, Didier R, Favereau X, Souteyrand G, Ohlmann P, Doisy V, Grollier G, Gommeaux A, Claudel JP, Bourlon F, Bertrand B, Laskar M, Iung B, Gilard M, Laskar M, Eltchaninoff H, Fajadet J, Iung B, Teiger E, Donzeau-Gouge P, Leprince P, Leguerrier A, Prat A, Chevreul K, Lievre M, Bertrand M, Cassagne J, Boschat J, Lusson JR, Mathieu P, Logeais Y, Bessou JP, Lefevre T, Chevalier B, Farge A, Garot P, Hovasse T, Morice MC, Romano M, Gouge PD, Tchetche D, Vahdat O, Farah B, Fajadet J, Carrie D, Dumonteil N, Fournial G, Marcheix B, Himbert D, Nataf P, Vahanian A, Albat B, Leclercq F, Piot C, Schmutz L, Aubas P, du Cailar A, Dubar A, Durrleman N, Fargosz F, Levy G, Maupas E, Rivalland F, Robert G, Eltchaninoff H, Bessou JP, Cribier A, Tron C, Sudre A, Juthier F, Modine T, Van Belle E, Banfi C, Blanchard D, Sallerin T, Bar O, Barbey C, Chassaing S, Chatel D, Le Page O, Tauran A, Rioufol G, Cao D, Dauphin R, Durand de Gevigney G, Finet G, Jegaden O, Obadia JF, Leprince P, Beygui F, Collet JP, Pavie A, Collet F, Pecheux, Bayet, Vaillant A, Vicat J, Wittenberg O, Houel R, Joly P, Rosario R, Bergeron P, Bille J, Gelisse R, Teiger E, Couetil JP, Dubois Rande JL, Hayat D, Fougeres E, Monin JL, Mouillet G, Dos Santos P, Arsac F, Choukroun E, Dijos M, Guibaud JP, Leroux L, Elia N, Meneveau N, Descotes G, Chocron S, Schiele F, Caussin C, Azmoun A, Ghostine S, Nottin R, Tirouvanziam A, Crochet D, Gaudin R, Roussel JC, Guyon P, Bonnet N, Digne F, Mesnidrey P, Royer T, Stratiev V, Grisoli D, Bonnet JL, Cuisset T, Grisoli D, Le Breton H, Abouliatim I, Bedossa M, Boulmier D, Verhoye JP, Delepine S, Debrux JL, Furber A, Pinaud F, Gilard M, Bezon E, Boschat J, Choplain JN, Favereau X, Bical O, Dambrin G, Deleuze P, Jegou A, Lusson JR, Azarnouch K, Durel N, Innorta A, Souteyrand G, Doisy V, Lienhart Y, Roriz R, Staat P, Blanchard D, Fabiani JN, Lafont A, Zegdi R, Heudes D, Ohlmann P, Kindo M, Mazzucotelli JP, Zupan M, Grollier G, Ivascau C, Lognone T, Massetti M, Sabatier R, Huret B, Gommeaux A, Hochart P, Pecheux, Claudel JP, Bouchayer D, Gabrielle F, Pelissier F, Tremeau G, Bourlon F, Dreyfus G, Eker A, Habib Y, Hugues N, Mialhe C, Bertrand B, Chavanon O, Porcu P, and Vanzetto G. 2016. Late Outcomes of Transcatheter Aortic Valve Replacement in High-Risk Patients: The FRANCE-2 Registry. *Journal of the American College of Cardiology* 68:1637-1647. 10.1016/j.jacc.2016.07.747

Godino C, Maisano F, Montorfano M, Latib A, Chieffo A, Michev I, Al-Lamee R, Bande M, Mussardo M, Arioli F, Ielasi A, Cioni M, Taramasso M, Arendar I, Grimaldi A, Spagnolo P, Zangrillo A, La Canna G, Alfieri O, and Colombo A. 2010. Outcomes after transcatheter aortic valve implantation with both Edwards-SAPIEN and CoreValve devices in a single center: the Milan experience. *JACC Cardiovasc Interv* 3:1110-1121. 10.1016/j.jcin.2010.09.012

Gotzmann M, Thiessen A, Lindstaedt M, Mügge A, and Ewers A. 2013. Left atrial diameter, aortic mean gradient, and hemoglobin for risk stratification in patients undergoing transcatheter aortic valve implantation. *Clinical Cardiology* 36:228-234. 10.1002/clc.22100

Hall PS, O'Donnell CI, Mathew V, Garcia S, Bavry AA, Banerjee S, Jneid H, Denktas AE, Giacomini JC, Grossman PM, Aggarwal K, Zimmet JM, Tseng EE, Gozdecki L, Burke L, Bertog SC, Buchbinder M, Plomondon ME, Waldo SW, and Shunk KA. 2019. Outcomes of Veterans Undergoing TAVR Within Veterans Affairs Medical Centers: Insights From the Veterans Affairs Clinical Assessment, Reporting, and Tracking Program. *JACC: Cardiovascular Interventions* 12:2186-2194. 10.1016/j.jcin.2019.04.040

Hayashida K, Romano M, Lefevre T, Chevalier B, Farge A, Hovasse T, Le Houerou D, and Morice MC. 2013. The transaortic approach for transcatheter aortic valve implantation: a valid alternative to the transapical access in patients with no peripheral vascular option. A single center experience. *European Journal of Cardio-thoracic Surgery* 44:692-700. <https://dx.doi.org/10.1093/ejcts/ezt037>

Hellhammer K, Piayda K, Afzal S, Kleinebrecht L, Makosch M, Hennig I, Quast C, Jung C, Polzin A, Westenfeld R, Kelm M, Zeus T, and Veulemans V. 2018. The Latest Evolution of the Medtronic CoreValve System in the Era of Transcatheter Aortic Valve Replacement: Matched Comparison of the Evolut PRO and Evolut R. *JACC Cardiovascular interventions* 11:2314-2322. 10.1016/j.jcin.2018.07.023

Henn MC, Percival T, Zajarias A, Melby SJ, Lindman BR, Quader N, Damiano RJ, Moon MR, Lasala JM, Rao RS, Bell J, Damiano MS, and Maniar HS. 2017. Learning Alternative Access Approaches for Transcatheter Aortic Valve Replacement: Implications for New Transcatheter Aortic Valve Replacement Centers. *Annals of Thoracic Surgery* 103:1399-1405. 10.1016/j.athoracsur.2016.08.068

Herrmann HC, Thourani VH, Kodali SK, Makkar RR, Szeto WY, Anwaruddin S, Desai N, Lim S, Malaisrie SC, Kereiakes DJ, Ramee S, Greason KL, Kapadia S, Babaliaros V, Hahn RT, Pibarot P, Weissman NJ, Leipsic J, Whisenant BK, Webb JG, MacK MJ, and Leon MB. 2016. One-Year Clinical Outcomes with SAPIEN 3 Transcatheter Aortic Valve Replacement in High-Risk and Inoperable Patients with Severe Aortic Stenosis. *Circulation* 134:130-140. 10.1161/CIRCULATIONAHA.116.022797

Iung B, Laouenan C, Himbert D, Eltchaninoff H, Chevreul K, Donzeau-Gouge P, Fajadet J, Leprince P, Leguerrier A, Lievre M, Prat A, Teiger E, Laskar M, Vahanian A, and Gilard M. 2014. Predictive factors of early mortality after transcatheter aortic valve implantation: individual risk assessment using a simple score. *Heart* 100:1016-1023. 10.1136/heartjnl-2013-305314

Jarrett CM, Pelletier M, Shah PB, and Kaneko T. 2017. “Double-Stick” Transsubclavian Transcatheter Aortic Valve Replacement With Use of a Balloon Expandable Valve: A Less Invasive Option for Alternative Access. *Annals of Thoracic Surgery* 104:e195-e197. 10.1016/j.athoracsur.2017.02.064

Kalra SS, Firoozi S, Blackman D, Rashid S, Davies S, Moat N, Yeh J, Dalby M, Kabir T, Khogali SS, Anderson RA, Groves PH, Mylotte D, Hildick-Smith D, Rampat R, Kovac J, Gunarathne A, Laborde JC, and Brecker SJ. 2016. Initial experience of a second-generation self-expanding transcatheter aortic valve: The United Kingdom and Ireland IMPLANTERS registry. *EuroIntervention*:326.

Kasapkara HA, Aslan AN, Ayhan H, Baştuǧ S, Süygün H, Keleş T, Durmaz T, and Bozkurt E. 2016. Trans-subclavian aortic valve replacement with various bioprosthetic valves: Single-center experience. *Turk Kardiyoloji Dernegi Arsivi* 44:582-589. 10.5543/tkda.2016.45774

Kawaguchi AT, Collet JP, Cluzel P, Makri R, Laali M, DeFrance C, Furuya H, Murakami A, and Leprince P. 2017. Preoperative Risk Levels and Vascular Access in Transcatheter Aortic Valve Implantation—A Single-Institute Analysis—. *Artificial Organs* 41:130-138. 10.1111/aor.12754

Kiefer P, Seeburger J, Noack T, Schroter T, Linke A, Schuler G, Haensig M, Vollroth M, Mohr FW, and Holzhey DM. 2015. The role of the heart team in complicated transcatheter aortic valve implantation: a 7-year single-centre experience. *European Journal of Cardio-thoracic Surgery* 47:1090-1096. <https://dx.doi.org/10.1093/ejcts/ezu379>

Kobayashi J, Shimahara Y, Fujita T, Kanzaki H, Amaki M, Hata H, Kume Y, Yamashita K, and Okada A. 2016. Early results of simultaneous transaortic transcatheter aortic valve implantation and total arterial off-pump coronary artery revascularization in high-risk patients. *Circulation Journal* 80:1946-1950. 10.1253/circj.CJ-16-0329

Kochman J, Rymuza B, Huczek Z, Kołtowski Ł, Ścisło P, Wilimski R, Ścibisz A, Stanecka P, Filipiak KJ, and Opolski G. 2016. Incidence, predictors and impact of severe periprocedural bleeding according to VARC-2 criteria on 1-year clinical outcomes in patients after transcatheter aortic valve implantation. *International Heart Journal* 57:35-40. 10.1536/ihj.15-195

Kodali S, Thourani VH, White J, Malaisrie SC, Lim S, Greason KL, Williams M, Guerrero M, Eisenhauer AC, Kapadia S, Kereiakes DJ, Herrmann HC, Babaliaros V, Szeto WY, Hahn RT, Pibarot P, Weissman NJ, Leipsic J, Blanke P, Whisenant BK, Suri RM, Makkar RR, Ayele GM, Svensson LG, Webb JG, Mack MJ, Smith CR, and Leon MB. 2016. Early clinical and echocardiographic outcomes after SAPIEN 3 transcatheter aortic valve replacement in inoperable, high-risk and intermediate-risk patients with aortic stenosis. *European Heart Journal* 37:2252-2262. 10.1093/eurheartj/ehw112

Koh EY, Lam KY, Bindraban NR, Cocchieri R, Planken RN, Koch KT, Baan J, Jr., de Mol BA, and Marquering HA. 2015. Aortic valve calcification as a predictor of location and severity of paravalvular regurgitation after transcatheter aortic valve implantation. *Interactive Cardiovascular & Thoracic Surgery* 20:345-350. <https://dx.doi.org/10.1093/icvts/ivu413>

Lange R, Bleiziffer S, Piazza N, Mazzitelli D, Hutter A, Tassani-Prell P, Laborde JC, and Bauernschmitt R. 2011. Incidence and treatment of procedural cardiovascular complications associated with trans-arterial and trans-apical interventional aortic valve implantation in 412 consecutive patients. *European Journal of Cardio-thoracic Surgery* 40:1105-1113. 10.1016/j.ejcts.2011.03.022

Lardizabal JA, O'Neill BP, Desai HV, Macon CJ, Rodriguez AP, Martinez CA, Alfonso CE, Bilsker MS, Carillo RG, Cohen MG, Heldman AW, O'Neill WW, and Williams DB. 2013. The transaortic approach for transcatheter aortic valve replacement: initial clinical experience in the United States. *Journal of the American College of Cardiology* 61:2341-2345. <https://dx.doi.org/10.1016/j.jacc.2013.02.076>

López-Otero D, Muñoz-García AJ, Avanzas P, Lozano I, Alonso-Briales JH, Souto-Castro P, Morís C, Hernández-García JM, and Trillo-Nouche R. 2011. Axillary approach for transcatheter aortic valve implantation: Optimization of the endovascular treatment for the aortic valve stenosis. *Revista Espanola de Cardiologia* 64:121-126. 10.1016/j.recesp.2010.08.004

Lucchese G, Montarello N, and Bapat V. 2017. Modified Single-Puncture Technique for Transcatheter Aortic Valve Implantation in Patients without Peripheral Vascular Access. *Thoracic and Cardiovascular Surgeon* 65:231-233. 10.1055/s-0036-1586494

Mathur M, Krishnan SK, Levin D, Aldea G, Reisman M, and McCabe JM. 2017. A Step-by-Step Guide to Fully Percutaneous Transaxillary Transcatheter Aortic Valve Replacement. *Structural Heart* 1:209-215. 10.1080/24748706.2017.1370156

McCarthy FH, Spragan DD, Savino D, Dibble T, Hoedt AC, McDermott KM, Bavaria JE, Herrmann HC, Anwaruddin S, Giri J, Szeto WY, Groeneveld PW, and Desai ND. 2017. Outcomes, readmissions, and costs in transfemoral and alterative access transcatheter aortic valve replacement in the US Medicare population. *Journal of Thoracic and Cardiovascular Surgery* 154:1224-1232.e1221. 10.1016/j.jtcvs.2017.04.090

Modine T, Obadia JF, Choukroun E, Rioufoul G, Sudre A, Laborde JC, and Leprince P. 2011. Transcutaneous aortic valve implantation using the axillary/subclavian access: Feasibility and early clinical outcomes. *Journal of Thoracic and Cardiovascular Surgery* 141:487-491. 10.1016/j.jtcvs.2010.01.044

Modine T, Sudre A, Collet F, Delhaye C, Lemesles G, Fayad G, and Koussa M. 2012. Transcutaneous aortic valve implantation using the axillary/subclavian access with patent left internal thoracic artery to left anterior descending artery: Feasibility and early clinical outcomes. *Journal of Thoracic and Cardiovascular Surgery* 144:1416-1420. 10.1016/j.jtcvs.2012.01.031

O'Hair DP, Bajwa TK, Popma JJ, Watson DR, Yakubov SJ, Adams DH, Sharma S, Robinson N, Petrossian G, Caskey M, Byrne T, Kleiman NS, Zhang A, and Reardon MJ. 2018. Direct Aortic Access for Transcatheter Aortic Valve Replacement Using a Self-Expanding Device. *Annals of Thoracic Surgery* 105:484-490. 10.1016/j.athoracsur.2017.07.051

Parma R, Dabrowski M, Ochala A, Witkowski A, Dudek D, Siudak Z, and Legutko J. 2017. The Polish Interventional Cardiology TAVI Survey (PICTS): Adoption and practice of transcatheter aortic valve implantation in Poland. *Postepy w Kardiologii Interwencyjnej* 13:10-17. 10.5114/aic.2017.66181

Pascual I, Avanzas P, Munoz-Garcia AJ, Lopez-Otero D, Jimenez-Navarro MF, Cid-Alvarez B, del Valle R, Alonso-Briales JH, Ocaranza-Sanchez R, Alfonso F, Hernandez JM, Trillo-Nouche R, and Moris C. 2013. Percutaneous implantation of the CoreValve(R) self-expanding valve prosthesis in patients with severe aortic stenosis and porcelain aorta: medium-term follow-up. *Rev Esp Cardiol (Engl Ed)* 66:775-781. 10.1016/j.rec.2013.03.001

Patel SV, Jhamnani S, Patel P, Sonani R, Savani C, Patel N, Patel NJ, Panaich SS, Patel M, Theodore S, Grines C, and Badheka AO. 2016. Influence of same-day admission on outcomes following transcatheter aortic valve replacement. *Journal of Cardiac Surgery* 31:608-616. 10.1111/jocs.12819

Petronio AS, De Carlo M, Bedogni F, Maisano F, Ettori F, Klugmann S, Poli A, Marzocchi A, Santoro G, Napodano M, Ussia GP, Giannini C, Brambilla N, and Colombo A. 2012. 2-year results of CoreValve implantation through the subclavian access: A propensity-matched comparison with the femoral access. *Journal of the American College of Cardiology* 60:502-507. 10.1016/j.jacc.2012.04.014

Petronio AS, De Carlo M, Bedogni F, Marzocchi A, Klugmann S, Maisano F, Ramondo A, Ussia GP, Ettori F, Poli A, Brambilla N, Saia F, De Marco F, and Colombo A. 2010. Safety and efficacy of the subclavian approach for transcatheter aortic valve implantation with the CoreValve revalving system. *Circulation: Cardiovascular Interventions* 3:359-366. 10.1161/CIRCINTERVENTIONS.109.930453

Porterie J, Mayeur N, Lhermusier T, Dumonteil N, Chollet T, Lairez O, and Marcheix B. 2019. Aortic and innominate routes for transcatheter aortic valve implantation. *Journal of Thoracic and Cardiovascular Surgery* 157:1393-1401.e1397. 10.1016/j.jtcvs.2018.07.098

Reardon MJ, Adams DH, Coselli JS, Deeb GM, Kleiman NS, Chetcuti S, Yakubov SJ, Heimansohn D, Hermiller J, Hughes GC, Harrison JK, Khabbaz K, Tadros P, Zorn GL, Merhi W, Heiser J, Petrossian G, Robinson N, Maini B, Mumtaz M, Lee JS, Gleason TG, Resar J, Conte J, Watson D, Chenoweth S, and Popma JJ. 2014. Self-expanding transcatheter aortic valve replacement using alternative access sites in symptomatic patients with severe aortic stenosis deemed extreme risk of surgery. *Journal of Thoracic and Cardiovascular Surgery* 148:2869-2876. 10.1016/j.jtcvs.2014.07.020

Rogers T, Waksman R, Harrison JK, Deeb GM, Zhang AQ, Hermiller JB, Jr., Popma JJ, and Reardon MJ. 2018. Impact of Balloon Predilatation on Hemodynamics and Outcomes After Transcatheter Aortic Valve Implantation With the Self-Expanding CoreValve Prosthesis. *The American journal of cardiology* 121:1358-1364. 10.1016/j.amjcard.2018.02.008

Romano M, Frank D, Cocchieri R, Jagielak D, Bonaros N, Aiello M, Lapeze J, Laine M, Chocron S, Muir D, Eichinger W, Thielmann M, Labrousse L, Arne Rein K, Verhoye JP, Gerosa G, Baumbach H, Deutsch C, Bramlage P, Thoenes M, and Bapat V. 2017. Transaortic transcatheter aortic valve implantation using SAPIEN XT or SAPIEN 3 valves in the ROUTE registry. *Interactive Cardiovascular and Thoracic Surgery* 25:757-764. 10.1093/icvts/ivx159

Saia F, Ciuca C, Taglieri N, Marrozzini C, Savini C, Bordoni B, Dall'Ara G, Moretti C, Pilato E, Martin-Suarez S, Petridis FD, Di Bartolomeo R, Branzi A, and Marzocchi A. 2013. Acute kidney injury following transcatheter aortic valve implantation: incidence, predictors and clinical outcome. *Int J Cardiol* 168:1034-1040. 10.1016/j.ijcard.2012.10.029

Salizzoni S, D'Onofrio A, Agrifoglio M, Colombo A, Chieffo A, Cioni M, Besola L, Regesta T, Rapetto F, Tarantini G, Napodano M, Gabbieri D, Saia F, Tamburino C, Ribichini F, Cugola D, Aiello M, Sanna F, Iadanza A, Pompei E, Stefano P, Cappai A, Minati A, Cassese M, Martinelli GL, Agostinelli A, Fiorilli R, Casilli F, Reale M, Bedogni F, Petronio AS, Mozzillo RA, Bonmassari R, Briguori C, Liso A, Sardella G, Bruschi G, Fiorina C, Filippini C, Moretti C, D'Amico M, La Torre M, Conrotto F, Di Bartolomeo R, Gerosa G, and Rinaldi M. 2016. Early and mid-term outcomes of 1904 patients undergoing transcatheter balloon-expandable valve implantation in Italy: results from the Italian Transcatheter Balloon-Expandable Valve Implantation Registry (ITER). *Eur J Cardiothorac Surg* 50:1139-1148. 10.1093/ejcts/ezw218

Sannino A, Stoler RC, Lima B, Szerlip M, Henry AC, Vallabhan R, Kowal RC, Brown DL, Mack MJ, and Grayburn PA. 2016. Frequency of and Prognostic Significance of Atrial Fibrillation in Patients Undergoing Transcatheter Aortic Valve Implantation. *American Journal of Cardiology* 118:1527-1532. 10.1016/j.amjcard.2016.08.017

Schäfer U, Ho Y, Frerker C, Schewel D, Sanchez-Quintana D, Schofer J, Bijuklic K, Meincke F, Thielsen T, Kreidel F, and Kuck KH. 2012. Direct percutaneous access technique for transaxillary transcatheter aortic valve implantation: "the Hamburg Sankt Georg approach". *JACC: Cardiovascular Interventions* 5:477-486. 10.1016/j.jcin.2011.11.014

Scherner M, Madershahian N, Ney S, Kuhr K, Rosenkranz S, Rudolph TK, Kuhn E, Slottosch I, Deppe A, Choi YH, Baldus S, and Wahlers T. 2015. Focus on the surgical approach to transcatheter aortic valve implantation: Complications, outcome, and preoperative risk adjustment. *Journal of Thoracic & Cardiovascular Surgery* 150:841-849. <https://dx.doi.org/10.1016/j.jtcvs.2015.06.030>

Sherif MA, Zahn R, Gerckens U, Sievert H, Eggebrecht H, Hambrecht R, Sack S, Richardt G, Schneider S, Senges J, and Brachmann J. 2014. Effect of gender differences on 1-year mortality after transcatheter aortic valve implantation for severe aortic stenosis: Results from a multicenter real-world registry. *Clinical Research in Cardiology* 103:613-620. 10.1007/s00392-014-0690-6

Sinning JM, Horack M, Grube E, Gerckens U, Erbel R, Eggebrecht H, Zahn R, Linke A, Sievert H, Figulla HR, Kuck KH, Hauptmann KE, Hoffmann E, Hambrecht R, Richardt G, Sack S, Senges J, Nickenig G, and Werner N. 2012. The impact of peripheral arterial disease on early outcome after transcatheter aortic valve implantation: results from the German Transcatheter Aortic Valve Interventions Registry. *Am Heart J* 164:102-110 e101. 10.1016/j.ahj.2012.04.016

Stańska A, Jagielak D, Kowalik M, Brzeziński M, Pawlaczyk R, Fijałkowska J, Karolak W, Rogowski J, and Bramlage P. 2018. Health-related quality of life following transcatheter aortic valve implantation using transaortic, transfemoral approaches and surgical aortic valve replacement—a single-center study. *Journal of Geriatric Cardiology* 15:657-665. 10.11909/j.issn.1671-5411.2018.11.002

Suri RM, Gulack BC, Brennan JM, Thourani VH, Dai D, Zajarias A, Greason KL, Vassileva CM, Mathew V, Nkomo VT, Mack MJ, Rihal CS, Svensson LG, Nishimura RA, O'Gara PT, and Holmes DR, Jr. 2015. Outcomes of Patients With Severe Chronic Lung Disease Who Are Undergoing Transcatheter Aortic Valve Replacement. *Annals of Thoracic Surgery* 100:2136-2145; discussion 2145-2136. <https://dx.doi.org/10.1016/j.athoracsur.2015.05.075>

Taramasso M, Maisano F, Cioni M, Denti P, Godino C, Montorfano M, Colombo A, and Alfieri O. 2011. Trans-apical and trans-axillary percutaneous aortic valve implantation as alternatives to the femoral route: Short- and middle-term results. *European Journal of Cardio-thoracic Surgery* 40:49-55. 10.1016/j.ejcts.2010.11.039

Tayal R, Hawatmeh A, Thawabi M, Haik B, Wasty N, and Russo M. 2017. Percutaneous Transaxillary Transcatheter Aortic Valve Replacement. *The Journal of invasive cardiology* 29:E72-E73.

Thourani VH, Gunter RL, Neravetla S, Block P, Guyton RA, Kilgo P, Lerakis S, Devireddy C, Leshnower B, Mavromatis K, Stewart J, Simone A, Keegan P, Nguyen TC, Merlino J, and Babaliaros V. 2013. Use of transaortic, transapical, and transcarotid transcatheter aortic valve replacement in inoperable patients. *Annals of Thoracic Surgery* 96:1349-1357. <https://dx.doi.org/10.1016/j.athoracsur.2013.05.068>

Thourani VH, Li C, Devireddy C, Jensen HA, Kilgo P, Leshnower BG, Mavromatis K, Sarin EL, Nguyen TC, Kanitkar M, Guyton RA, Block PC, Maas AL, Simone A, Keegan P, Merlino J, Stewart JP, Lerakis S, and Babaliaros V. 2015. High-risk patients with inoperative aortic stenosis: use of transapical, transaortic, and transcarotid techniques. *Annals of Thoracic Surgery* 99:817-823; discussion 823-815. <https://dx.doi.org/10.1016/j.athoracsur.2014.10.012>

Watanabe Y, Hayashida K, Lefevre T, Chevalier B, Hovasse T, Romano M, Garot P, Farge A, Donzeau-Gouge P, Bouvier E, Cormier B, and Morice MC. 2013. Is EuroSCORE II better than EuroSCORE in predicting mortality after transcatheter aortic valve implantation? *Catheterization & Cardiovascular Interventions* 81:1053-1060. <https://dx.doi.org/10.1002/ccd.24702>

Wenaweser P, Stortecky S, Heg D, Tueller D, Nietlispach F, Falk V, Pedrazzini G, Jeger R, Reuthebuch O, Carrel T, Raber L, Amann FW, Ferrari E, Toggweiler S, Noble S, Roffi M, Gruenenfelder J, Juni P, Windecker S, and Huber C. 2014. Short-term clinical outcomes among patients undergoing transcatheter aortic valve implantation in Switzerland: the Swiss TAVI registry. *EuroIntervention* 10:982-989. 10.4244/eijv10i8a166
