# Supplementary material for: Direct aortic route versus transaxillary route for transcatheter aortic valve replacement: a systematic review and meta-analysis
Source: PeerJ. 2020 May 12;8:e9102. doi: 10.7717/peerj.9102 (PMC7227658; doi:10.7717/peerj.9102)
Supplement: Supplemental Information 6 [file peerj-08-9102-s006.docx]

**Supplementary Table 3.** Newcastle-Ottawa Scale quality assessment of included studies.

|  | Selection | | | | Comparability | Outcome | | |  |
| --- | --- | --- | --- | --- | --- | --- | --- | --- | --- |
| First author, year | Representativeness of the exposed cohort | Selection of the non-exposed cohort | Ascertainment of exposure | Outcome of interest not present at start of study | Comparability | Assessment of outcome | Was follow-up long enough for outcomes to occur? | Adequacy of follow -up of cohorts | Total |
| Khan, 2018 | ★ |  | ★ | ★ | ★ | ★ | ★ | ★ | 7 |
| Damluji, 2018 | ★ |  | ★ | ★ | ★ | ★ | ★ | ★ | 7 |
| Fiorina, 2016 | ★ |  | ★ | ★ | ★ | ★ | ★ | ★ | 7 |
| Adamo, 2015 | ★ |  | ★ | ★ | ★ | ★ | ★ | ★ | 7 |
| Zhan, 2020 | ★ |  | ★ | ★ |  | ★ | ★ | ★ | 6 |
| Dahle, 2019 | ★ |  | ★ | ★ |  | ★ | ★ | ★ | 6 |
| Hysi, 2019 | ★ |  | ★ | ★ |  | ★ | ★ | ★ | 6 |
| Gleason, 2018 | ★ |  | ★ | ★ |  | ★ | ★ | ★ | 6 |
| Terzian, 2017 | ★ |  | ★ | ★ |  | ★ | ★ | ★ | 6 |
| Schäfer, 2017 | ★ |  | ★ | ★ |  | ★ | ★ | ★ | 6 |
| Laflamme, 2014 | ★ |  | ★ | ★ |  | ★ | ★ | ★ | 6 |
| Muensterer, 2013 | ★ |  | ★ | ★ |  | ★ | ★ | ★ | 6 |
| Testa, 2012 | ★ |  | ★ | ★ |  | ★ | ★ | ★ | 6 |
| Gilard, 2012 | ★ |  | ★ | ★ |  | ★ | ★ | ★ | 6 |
| Romano, 2019 | ★ |  | ★ | ★ |  | ★ | ★ | ★ | 6 |
| Cocchieri, 2019 |  |  | ★ | ★ |  | ★ | ★ | ★ | 5 |
| D’Ancona, 2019 | ★ |  | ★ | ★ |  | ★ | ★ | ★ | 6 |
| Petzina, 2017 | ★ |  | ★ | ★ |  | ★ | ★ | ★ | 6 |
| Bruschi, 2017 | ★ |  | ★ | ★ |  | ★ | ★ | ★ | 6 |
| Bonaros, 2017 |  |  | ★ | ★ |  | ★ | ★ | ★ | 5 |
| Ropponen, 2016 | ★ |  | ★ | ★ |  | ★ | ★ | ★ | 6 |
| Arai, 2016 | ★ |  | ★ | ★ |  | ★ | ★ | ★ | 6 |
| Wendt, 2015 | ★ |  | ★ | ★ |  | ★ | ★ | ★ | 6 |
| Thourani, 2015 | ★ |  | ★ | ★ |  | ★ | ★ | ★ | 6 |
| Ribeiro, 2015 | ★ |  | ★ | ★ |  | ★ | ★ | ★ | 6 |
| Ramlawi, 2015 | ★ |  | ★ | ★ |  | ★ | ★ | ★ | 6 |
| Okuyama, 2015 | ★ |  | ★ | ★ |  | ★ | ★ | ★ | 6 |
| Jagielak, 2015 | ★ |  | ★ | ★ |  | ★ | ★ | ★ | 6 |
| Bruschi, 2015 | ★ |  | ★ | ★ |  | ★ | ★ | ★ | 6 |
| Spargias, 2014 | ★ |  | ★ | ★ |  | ★ | ★ | ★ | 6 |
| Dahle, 2014 | ★ |  | ★ | ★ |  | ★ | ★ | ★ | 6 |
